# Cracking Ali Baba’s code

**DOI:** 10.7554/eLife.28600

**Published:** 2017-06-14

**Authors:** Oliver Billker

**Affiliations:** Wellcome Trust Sanger Institute, Hinxton, United Kingdomoliver.billker@sanger.ac.uk

**Keywords:** malaria, sporozoite, P. vivax, P. berghei, Hepatocyte, P. yoelii, Human, Mouse, *P. falciparum*, Other

## Abstract

A protein called P36 holds the key to how different species of malaria parasite invade liver cells.

**Related research article** Manzoni G, Marinach C, Topçu S, Briquet S, Grand M, Tolle M, Gransagne M, Lescar J, Andolina C, Franetich JF, Zeisel MB, Huby T, Rubinstein E, Snounou G, Mazier D, Nosten F, Baumert TF, Silvie O. 2017. *Plasmodium* P36 determines host cell receptor usage during sporozoite invasion. *eLife* **6**:e25903. doi: 10.7554/eLife.25903

“Open sesame!” These are the magic words through which Ali Baba and the 40 thieves gain access to a treasure cave. The malaria parasite is a type of thief that seeks to exploit the riches of a different hiding place. Now, in eLife, Olivier Silvie of Sorbonne University and colleagues – including Giulia Manzoni as first author – report that they have deciphered some of the code that these parasites use to gain entry into cells in the liver (Manzoni et al., 2017).

Malaria is caused by *Plasmodium* parasites, which are deposited into the skin through the bites of infected mosquitoes. Inside the body, the parasites first have to find and gain entry to liver cells. For the first few days of an infection the liver provides a perfect hiding place, being somewhere the parasites can use the plentiful nutrients inside the cells to develop quickly. A single parasite can exploit this niche to grow and divide rapidly. In this way, it can produce enough parasites to overwhelm the immune system once they are released into the blood stream. Although the liver phase does not cause malaria (the disease is caused by the parasites replicating inside red blood cells), it is important because it allows some species of *Plasmodium* to survive unnoticed in the human body for years, in a persistent state that can be difficult to treat. However, the liver stage parasites can also be targeted by vaccines.

How malaria parasites recognise and enter liver cells is poorly understood. At least two proteins – called CD81 and SR-B1 – on the surface of liver cells are thought to play a role (Silvie et al., 2003; Rodrigues et al., 2008). However, previous experiments with different *Plasmodium* species had yielded contradictory and confusing results. Manzoni et al. – who are based at several institutions in France, Thailand and the United Kingdom – now revisit this question by comparing four different species of malaria parasite. Two of these, called *Plasmodium falciparum* and *Plasmodium vivax*, infect humans; the other two, called *Plasmodium yoelii* and *Plasmodium berghei*, infect rodents.

In the experiments, the parasites attempted to invade a panel of host cells, which had been genetically modified so that they expressed either CD81, SR-B1 or both. The picture that emerged shows that the two species of *Plasmodium* that infect humans differ in the pathways they use to enter liver cells: *P. falciparum* uses CD81, whereas *P. vivax* uses the SR-B1 pathway. However, *P. berghei* can use both pathways (Figure 1). The same two liver cell surface proteins exploited by malaria parasites are also used by the hepatitis C virus to enter human liver cells (Bartosch et al., 2003).

**Figure 1.**
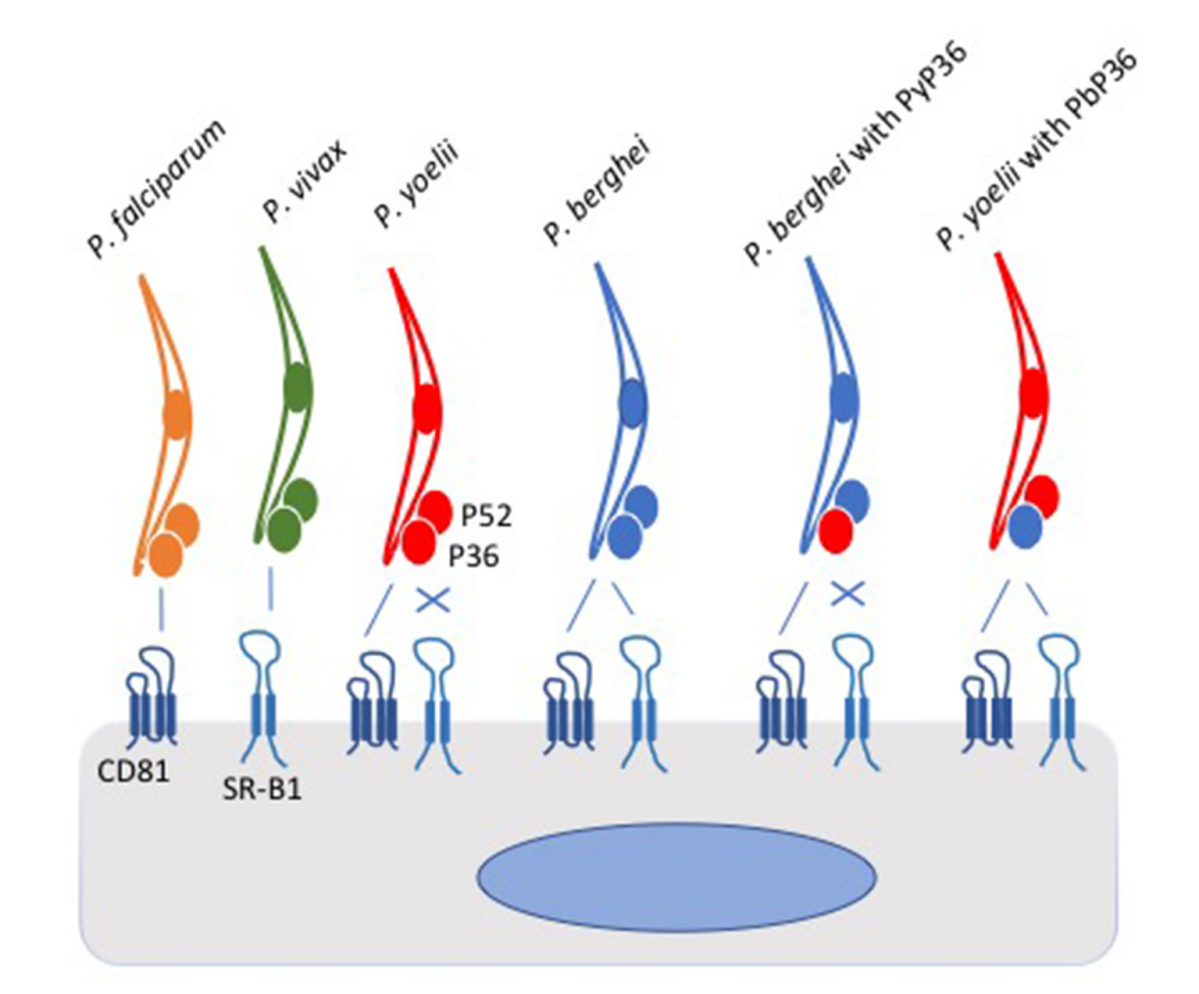
Schematic of *Plasmodium* parasites encountering a liver cell. Two proteins on the surface of the liver cell, called CD81 and SR-B1, are used by the parasites to invade the cell. However, parasite species differ in which of the pathways they can use. *P. falciparum* (yellow) and *P. yoelii* (red) interact with CD81 to enter the liver cell, *P. vivax* (green) interacts with SR-B1, and *P. berghei* (blue) can use either protein. Two proteins called P52 and P36 on the surface of the parasite were thought to play roles in the cell invasion process. Manzoni et al. found that the P36 protein on the surface of *P. berghei* holds the key to the ability of this parasite species to use the SR-B1 pathway. Replacing the P36 protein on *P. berghei* with the P36 protein from *P. yoelii* (PyP36) meant that the parasite could now only enter the liver cell via the CD81 pathway (as shown second from right). Conversely, replacing the P36 protein on *P. yoelii* with the P36 protein from *P. berghei* (PbP36) enabled this parasite species to use either CD81 or SR-B1 to enter liver cells (as shown far right). It remains to be seen whether P36 and SR-B1 interact directly. SR-B1: Scavenger Receptor B1.

Having described what seems to be part of the lock that opens the host cell, Manzoni et al. then searched for a protein in the parasite that may hold the key. Many parasite proteins are part of the molecular machinery that allows malaria parasites to enter cells (Bargieri et al., 2014), but how the parasites manage to target liver cells specifically is less well understood. Two proteins that may be involved in this process are P36 and P52, both of which the parasite can secrete onto its surface (Labaied et al., 2007). By removing these genes from the genome of the two species of *Plasmodium* that infect rodents, Manzoni et al. show that both proteins are required for parasites to invade liver cells.

Intriguingly, swapping around genes between parasite species revealed that *P. berghei* requires its unique form of P36 in order to use the SR-B1 pathway. Normally, *P. yoelii* cannot use the SR-B1 pathway to enter cells. However, *P. yoelii* was able to use the SR-B1 pathway if its P36 was swapped with the version of P36 used by *P. berghei* (which is able to use the SR-B1 pathway). This raises the possibility that P36 may interact directly with SR-B1.

Every child knows the code for Ali Baba’s treasure cave, but how the magic works remains Scheherazade’s secret. Now that we know some of the code that malaria parasites use to get into liver cells, we can investigate the mechanisms behind the invasion. It will be important to look for direct interactions between parasite proteins and host proteins. How do P36 and P52 interact with each other and with the parasite’s invasion machinery? Do host proteins play an active part through the signals they can send into the host cell? It will be particularly interesting to see how the small differences found in the gene sequence that encodes P36 in different species can open up an entirely new invasion pathway.
